# Editorial: Opportunistic pathogens: pathogenesis and multi-drug resistance mechanisms

**DOI:** 10.3389/fmicb.2025.1597769

**Published:** 2025-04-03

**Authors:** Shicheng Chen, Lisheng Liao, Mingxi Wang

**Affiliations:** ^1^Medical Laboratory Sciences Program, College of Health and Human Sciences, Northern Illinois University, DeKalb, IL, United States; ^2^Guangdong Province Key Laboratory of Microbial Signals and Disease Control, Integrative Microbiology Research Centre, South China Agricultural University, Guangzhou, China; ^3^Yun Leung Laboratory for Molecular Diagnostics, School of Medicine, Huaqiao University, Xiamen, Fujian, China

**Keywords:** opportunistic pathogens, multi-drug resistant, resistance mechanisms, host defense, horizontal gene transfer, regulatory systems

Opportunistic infections have long been underestimated in clinical settings, as the responsible pathogens are often dismissed as normal flora or harmless environmental organisms. Opportunistic pathogens are caplable of causing severe infections in immunocompromised individuals, even leading to significant outbreaks in healthcare settings. The management of opportunistic infections remains particularly challenging due to the absence of standardized guidelines for antibiotic susceptibility testing and the rising prevalence of multidrug resistance. In this Research Topic, titled “*Opportunistic pathogens: pathogenesis and multi-drug resistance mechanisms*”, we received 16 submissions and accepted nine manuscripts. Among these works, 8 original research explored pathogenesis and antimicrobial resistance mechanisms in *P. aeruginosa, Morganella morganii, Staphylococcus epidermidis* and *Klebsiella aerogenes, Mycobacterium tuberculosis*, and *Cryptococcus neoformans*. In addition, one manuscript reviewed roles of sterol metabolism in antifungal drug resistance.

The emerging carbapenem-resistant *Pseudomonas aeruginosa* (CRPA) poses a serious global health concern. Xie et al. analyzed 167 isolates in Fujian, China (2019–2021), revealing 58.7% antibiotic resistance and 70 CRPA cases. Molecular typing identified 46 sequence types, including two high-risk clones (ST1971 and ST357). Carbapenemase production was found in 22.9% of CRPA, mainly metallo-β-lactamases (MBLs), alongside *oprD* and *opdP* mutations. Increased biofilm formation and multidrug efflux pump gene expression were also observed. Zhao et al. screened and 143 clinical isolates of *P. aeruginosa* between 2021 and 2023 and found that at least 71 CRPA exhibiting a high carbapenem resistance. They further conducted genome analysis and predicted resistance genes in these CRPA isolates and identified a total of 54 resistance genes. They reported two novel metallo-β-lactamase genes (VIM-92 and blaVIM-24) discovered in this study. *bla*_VIM − 92_ was embedded in class 1 integrons within the Tn1403 transposon and found on a plasmid. Saha et al. investigated the coexistence of multidrug resistance and virulence factors in nosocomial *P. aeruginosa* strains. The isolates showed 70–75% susceptibility to aminoglycosides, 30–35% to quinolones, and lower rates for other antibiotics. Metallo-β-lactamase genes were found in 74.1% of strains. Additionally, 89% exhibited hemolysis, 80–90% produced pigments, and 46% formed strong biofilms. All displayed motility, and key virulence genes were detected in 60–80% of isolates.

Yang et al. studied the distribution of promoter types in class 1 integrons (intI1) in *Morganella morganii* clinical isolates and their role in regulating drug resistance gene expression. Among 97 isolates, 28.9% were positive for class 1 integrons, with 24.7% carrying gene cassettes that conferred resistance to aminoglycosides and trimethoprim. Three types of promoters (PcH1, PcS, and PcW) were identified, with P2 promoters found inactive. Stronger promoters led to increased expression of resistance genes, while a negative correlation between integrase (intI1) expression and promoter strength was observed, highlighting the regulatory role of these promoters in resistance.

Rodrigues et al. analyzed 10 *Klebsiella aerogenes* strains from ICU patients in a Brazilian hospital. Their results revealed that these strains exhibited the complete resistance to β-lactam antibiotics, including carbapenems, with varying resistance to aminoglycosides, quinolones, and tigecycline. Half of *K. aerogenes* strains were classified as multidrug-resistant, harboring β-lactamases genes such as *blaKPC-2* and *blaNDM-1*. A comprehensive genomic characterization of strain CRK317, which co-harbored *blaKPC-2* and *blaNDM-1*, identified numerous resistance determinants, including β-lactamases (*blaOXA-9, blaTEM-1, blaCTX-M-15*), aminoglycoside-modifying enzymes, and efflux pumps (*AcrA, tolC, mdtK*). Additionally, 22 genomic islands, insertion sequences, conjugative elements, and prophage regions were detected, suggesting their role in resistance gene dissemination. The presence of integrative and conjugative elements with a type IV secretion system further supports the horizontal transfer of resistance traits.

Lépine et al. investigated a linezolid-resistant *Staphylococcus epidermidis* (LRSE) outbreak at Tours University Hospital in France (2017–2021). Among 34 LRSE isolates, 20 were analyzed for antimicrobial susceptibility, genetic resistance mechanisms, and clonal relationships. All selected strains showed high-level linezolid resistance as well as multidrug resistance. G2576T mutation in the 23S rRNA may confer to the linezolid resistance, with no *cfr* gene detected. PFGE and MLST revealed 95% of strains belonged to ST2. Moreover, linezolid exposure was reported in 70% of patients, though no direct cross-transmission was found.

Singh and Dutta identified a virulence-associated small RNA (sRNA), MTS1338, that drove drug efflux in *Mycobacterium tuberculosis*. Rifampicin exposure increased MTS1338 expression over fourfold, enhancing bacterial growth under treatment. MTS1338 upregulated the efflux protein CydC by stabilizing its mRNA, reducing intracellular drug accumulation. Drug efflux assays confirmed that higher MTS1338 levels led to lower drug retention. These findings reveal a novel sRNA-driven regulatory mechanism contributing to drug resistance in *M. tuberculosis*, highlighting a potential target for developing more effective tuberculosis treatments.

Zheng et al. investigated genome plasticity as a mechanism of non-antifungal-induced antifungal resistance in *Cryptococcus neoformans*. This study further showed that tunicamycin, an ER stress inducer, affected aneuploidy formation in *Cryptococcus neoformans*. Both mild and severe ER stress induced aneuploid strains with diverse karyotypes, some showing resistance or cross-resistance to fluconazole and 5-flucytosine. These aneuploid strains also displayed genomic instability, losing extra chromosomes without stress. Tanwar et al. reviewed role of sterol metabolism in antifungal drug resistance. Sterols are crucial for eukaryotic cell membranes, affecting structure, function, and adaptability. Fungal sterol metabolism, including ergosterol, involves organelles like mitochondria and the ER, regulated by feedback mechanisms. Pathogenic fungi like *Candida, Aspergillus*, and *Cryptococcus* cause severe infections, especially in immunocompromised patients. Alterations in sterol metabolism and transport can contribute to antifungal resistance, emphasizing the challenges in treating these infections.

In summary, the nine articles included in this Research Topic cover multiple themes. For example, the rise of multidrug-resistant (MDR) pathogens, such as *Pseudomonas aeruginosa, Klebsiella aerogenes*, and *Staphylococcus epidermidis*, poses a significant global health threat. Moreover, studies in this Research Topic explored the pathogenesis, resistance mechanisms, and genetic factors behind these infections. Key findings include the identification of novel resistance genes, promoter types influencing gene expression in *Morganella morganii*, and the role of genome plasticity in antifungal resistance in *Cryptococcus neoformans*. Enhanced understanding of these mechanisms emphasizes the need for targeted treatments and antimicrobial stewardship.

